# Maternal psychological distress in the early postpartum period during COVID-19 pandemic: a pilot study

**DOI:** 10.1186/s12884-022-05166-0

**Published:** 2022-11-11

**Authors:** Yao-Sheng Wang, Wen-Hsin Cheng, I-Lun Chen, Hsin-Chun Huang

**Affiliations:** 1 Department of Pediatrics, Chiayi Chang Gung Memorial Hospital and Chang Gung University College of Medicine, Chiayi, Taiwan; 2 Department of Nursing, Kaohsiung Chang Gung Memorial Hospital and Chang Gung University College of Medicine, Kaohsiung, Taiwan; 3 Department of Pediatrics, Kaohsiung Chang Gung Memorial Hospital and Chang Gung University College of Medicine, Kaohsiung, Taiwan; 4 School of Traditional Chinese Medicine, College of Medicine, Chang Gung University, Linkou, Taiwan

**Keywords:** Stress, SARS-CoV-2, Saliva, Postpartum mother, Distress, Hospital regulation

## Abstract

**Background:**

The coronavirus disease 2019 infection (COVID-19) pandemic is a new global outbreak disease. According to the Taiwan Centers for Diseases Control statement, hospitals had to change their corresponding measures to prevent the spread of COVID-19. The frequency of parental visits to the special care nursery was reduced from three times to once daily. Visiting was not permitted from April 4 to May 10, 2020, and rooming-in with healthy neonates was discontinued, which could increase maternal postpartum distress. Therefore, this study was conducted to determine whether COVID-19 prevention increased maternal psychological distress.

**Methods:**

This prospective study used convenience sampling to enroll healthy mothers who had just delivered via normal spontaneous delivery. Based on the neonates’ status and visiting times, mothers were grouped into no-rooming-in, rooming-in, no-visiting, and one-visit/day groups. Mothers’ baseline characteristics were compared using the Chi-square or Fisher's exact test and *t*-test. Salivary cortisol levels and scores of Chinese versions of the Perceived Stress Scale (PSS) and State-Trait Anxiety Inventory were evaluated on postpartum days 1 and 3 and analyzed by one-way analysis of variance and a paired *t*-test.

**Results:**

There were 16, 58, 28, and 47 women categorized as no-rooming-in, rooming-in, no-visit, and one-visit/day groups, respectively. No significant differences were found between groups in mothers’ baseline characteristics and postpartum salivary cortisol levels. The PSS on day 3 was significantly higher than on day 1 in every group (*p* < 0.001). The PSS increasing trend in the no-rooming-in group was significantly greater than that in the no-visit group (*p* = 0.02) and significantly greater in the rooming-in group than that in the one-visit/day group (*p* = 0.001).

**Conclusion:**

Postpartum stress increased for all mothers and was an even more significant response to the COVID-19 pandemic than the stress associated with neonates’ hospitalization.

## Background

The coronavirus disease 2019 (COVID-19) pandemic started in December 2019 and had resulted in more than 3.7 million deaths as of June 2021, with a mortality rate of 2.15% worldwide [1]. The first confirmed case of COVID-19 was reported in Taiwan on January 21, 2020. The Central Epidemic Command Center established a prevention policy, including border control, reducing gatherings, maintaining social distance, measuring body temperature regularly, hand washing, and wearing masks, among other preventive measures [2]. According to the Taiwanese experience of severe acute respiratory syndrome (SARS) in 2003, the separation of suspected cases in advance can effectively protect people from the viral spread [3]. Thus, hospitals began to control access on February 14, 2020. Wearing a mask and measuring body temperature when entering the hospital became mandatory, although the cumulative confirmed local cases at that time in Taiwan were only 22 cases [4]. To protect the high-risk population from viral infection, the visiting time for the neonatal intensive care unit (NICU) was changed to once daily instead of three times daily. However, separating mothers and babies could cause mothers psychological stress.

In the general population, females compared with males and people aged younger than 40 years compared to who had older than 40 years had more anxiety about this new disease [5]. Other associated risks to have anxiety or psychological distress during the COVID-19 pandemic were women with a history of treatment for mental issues and those in an informal relationship in the first trimester of pregnancy [6]. They mainly worried about the threat of COVID-19 to mother and baby, inadequate prenatal care, and social isolation [7].

The prevalence of postpartum stress ranges widely from 1% to 30% [8]. The risk population, including maternal psychiatric history, history of trauma and adverse perinatal factors such as fear of childbirth, preterm birth, and preeclampsia, had a high prevalence of postpartum stress [8]. If mothers had preterm infants in NICU for a period and the hospital was far from their living places, it would result in less maternal-infant attachment, which would elevate stress levels and depressive symptoms [9, 10]. The COVID-19 pandemic acted as superimposed stress on mothers whose infants were in the NICU, leading to high levels of pediatric medical traumatic stress [11]. Postpartum stress is negatively associated with neonatal outcomes, including stunted growth and slow developmental progress [12]. Later in early childhood, poor language and cognitive development and poor gross and fine motor movement [12]. However, studies describing additional stress experienced by postpartum women during the COVID-19 pandemic in Taiwan are lacking.

The hypothalamic-pituitary-adrenal (HPA) axis of the neuroendocrine system encompasses the hypothalamus, the pituitary gland, and the adrenal glands, which interact directly [13]. Therefore, the neuroendocrine system regulates the human stress response. When a stressor occurs, glucocorticoid secretion from the adrenals is activated via the HPA axis. Plasma cortisol levels, including the protein-binding and free types of cortisol, respond directly to stress, but only the free type is transferred to saliva [14]. Thus, salivary cortisol level is not influenced by salivary concentration but correlates with the level of plasma cortisol and circadian rhythm [15]. Previous studies show that salivary cortisol levels are widely used to assess mothers’ stress during labor and predict the risk of postpartum psychosis [16–18].

Thus, we hypothesize that the COVID-19 prevention program would increase stress after delivery in the early postpartum stage. This study was conducted to understand postpartum stress experienced during the COVID-19 pandemic and the effects of the COVID-19 prevention program by using salivary cortisol assessment and the stress-related, self-reported questionnaires, Perceived Stress Scale (PSS) and the State-Trait Anxiety Inventory (STAI).

## Methods

### Participants

**Fig. 1:**
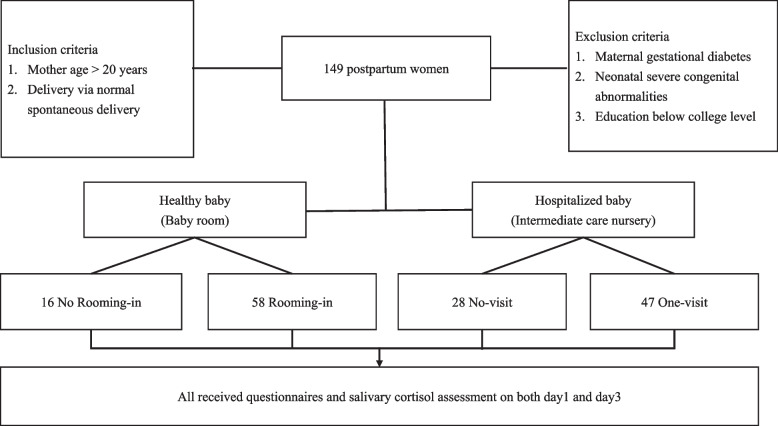
The flow diagram of enrolled participants

This study was conducted from May 1, 2020 to December 31, 2020. The inclusion criteria were mothers aged ≥ 20 years whose newborns were born via vaginal delivery at Chang Gung Memorial Hospital. Women with maternal gestational diabetes, neonatal severe congenital abnormalities, or education below college level were excluded. Education level was one of the criteria because the questionnaires can be understood at least a junior high school education. Mothers who met our criteria were enrolled after signing the informed consent. All methods were approved by the Institutional Review Board of hospital. Institutional Review Board number is 202000614B0A3. The ethical approval date was May 01, 2020. Enrolled mothers were divided into four groups and completed the assessment before discharge. Based on guidelines from the National Health Insurance, mothers with normal spontaneous delivery would be discharged from the hospital on the fourth day after giving birth. The total sample size was at least 76, which was calculated by G power with 0.8 power and 0.4 effect size. Mothers were grouped into no-rooming-in and rooming-in groups if their babies were in the nursery after delivery and no-visit and once-a-day visit groups if their babies were admitted to the special care nursery. Babies in the nursery regularly underwent a physical examination by pediatricians on the first and fourth days of life to ensure their health. Babies in the special care nursery typically had stable vital signs but may require oxygen support or need invasive examination even without symptoms, e.g., neonates from mothers with maternal autoimmune diseases, large caput succedaneum, hydronephrosis, and/or fever. The nursing staff cared for the neonates for 24 hours, and parents could only visit according to the ward schedule. Bottle feeding was given instead of breastfeeding because the neonates’ clinical diagnosis was not apparent during these three days after birth. In 2020, COVID-19 for neonates was not clearly understood, and the only diagnostic tool was polymerase chain reaction (PCR), which needs more than 24 hours. Some of the hospitalized babies had respiratory distress and we worried that they could be COVID-19 suspected cases. Thus, all the hospitalized neonates were isolated in incubators for the first three days of birth to prevent viral spread. The data were collected by structured questionnaires and physiological measurements from mothers on the first and third day after delivery. The flow diagram of enrolled participants was shown in Fig. 1.

### Subjective measures

#### Demographic and clinical characteristics

The questionnaire of baseline characteristics includes age, parity, education level, occupation and religion. Education level is divided into college and master’s degree or higher. The occupation included employment or not. Religion included theistic or not.

The Chinese version of PSS includes a total of 14 questions translated by Professor Li-Chuan Chu [19]. Cohen, Kamarck and Mermelstein designed the PSS in 1983 so that it could be easily understood by people with at least a junior high school education [20]. It is widely used to measure the extent to which some events are considered as stressful as others experienced within the last month. All items are summed from 0 to 56 points. Higher scores indicated greater perceived stress.

The STAI was designed by C.D. Spielberger in 1980 to measure feelings of apprehension, tension, nervousness, and worry [21]. This self-reported scale requires a sixth-grade reading level. Professors SK Chung and CF Long translated the Chinese version [22]. The scale consists of two parts, each designed with 20 questions to assess the level of anxiety associated with events (state anxiety) and personality (trait anxiety). The Chinese version of STAI had good multidimensional factorial validity [23]. State anxiety refers to the individual’s temporary emotional state. Nervousness and anxiety will change over time or may be relieved when the event disappears. Trait anxiety is long-term anxiety associated with a psychological tendency or personality traits. In some individuals, anxiety is often felt in response to a typical situation that everyone experiences daily. The total scores of the two scales are 20 and 80 points for each. Higher scores indicate a higher level of anxiety.

Saliva was collected from all mothers, at least 1 mL on the first and the third day after delivery. The questionnaires were filled out simultaneously. Because of the circadian rhythm in salivary cortisol, the collection time was fixed at 10:00 AM after gargling to reduce the effect. The mothers were advised to have breakfast before 8:30 AM and avoid high glycemic food. The samples were placed in the icebox and immediately sent to the laboratory. After removing mucus or other solid components by centrifuging, the samples were frozen at -20°C. The concentration of salivary cortisol was measured using the cortisol Elisa kit (kit number: 1-3002, Salimetrics, State College, PA, USA).

### Statistical analysis

Demographic data and outcome measures were expressed as mean and standard deviation for continuous variables, and frequency and percentage for categorical variables. Continuous and categorical variables were analyzed by one-way analysis of variance and Chi-square or Fisher's exact test, respectively. The scores of PSS, STAI, and salivary cortisol values on the first and third days were compared in two directions. First, compare them within groups on the first and third day after delivery. Second, compare them between groups of no-rooming-in group vs. no-visit group and rooming-in group vs. one-visit group. Paired *t*-test was used to analyze differences in the scores of PSS, STAI, and salivary cortisol values between the third and first days within each group. Linear regression analysis was used to calculate changing trends of significant scales between the four groups. All data were analyzed statistically using IBM SPSS 25 statistics software (IBM Corp., Armonk, NY, USA) and a *p*-value < 0.05 was considered statistically significant.

## Results

**Fig. 2:**
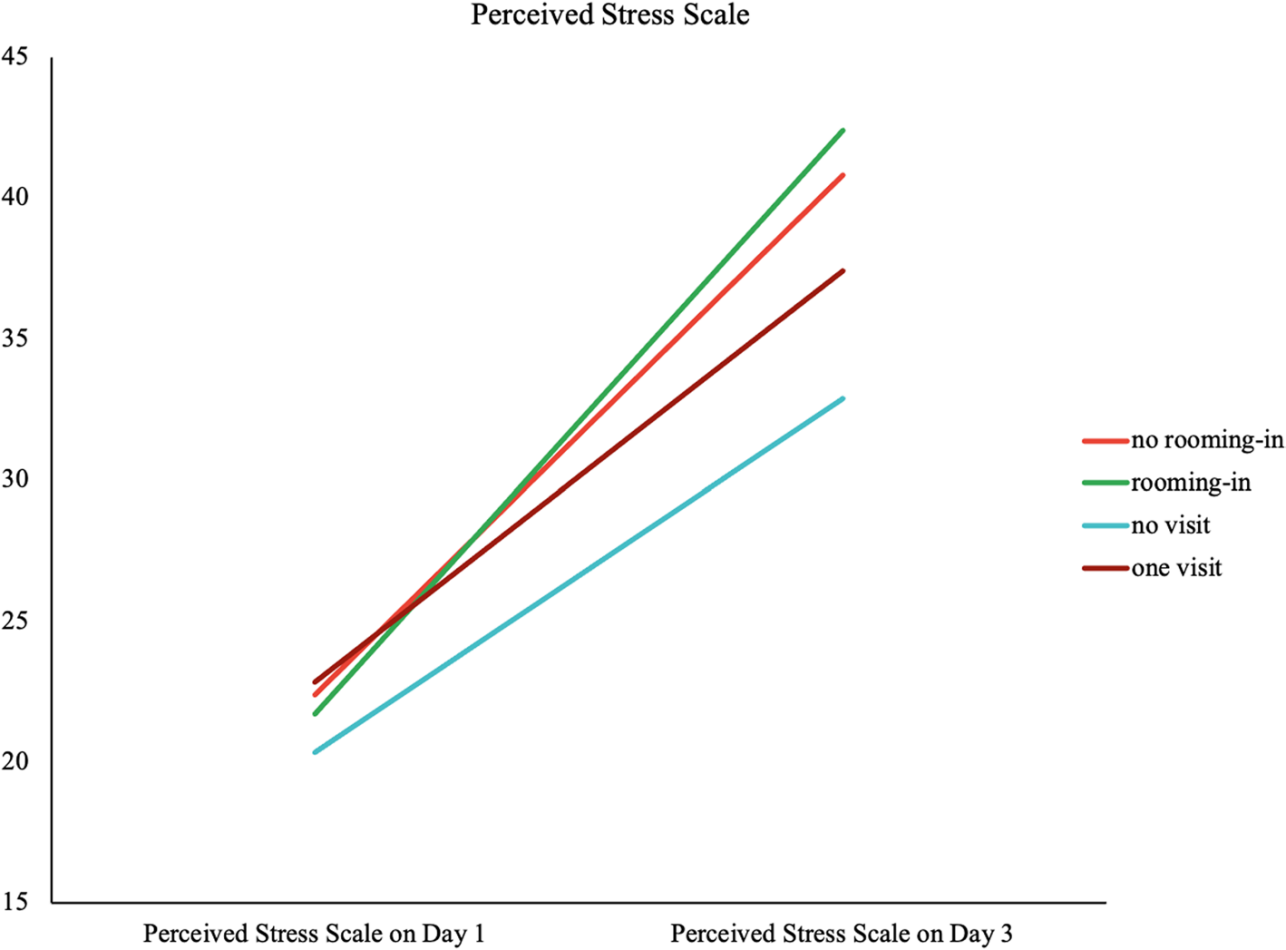
Perceived pressure scale increased on the third day compared with the first day in the four groups

A total of 149 postpartum women participated in this study; 16, 58, 28, and 47 mothers were in no-rooming-in, rooming-in, no-visit, and one-visit groups, respectively. No significant differences were noted in age, parity, education, occupation and religion between the four groups (Table 1). The Cronbach's alpha of PSS, STAI-state and STAI-trait on day 1 was 0.831, 0.826, and 0.823, respectively, and that on day 3 was 0.855, 0.834, and 0.816, respectively. No significant differences were also noted for PSS, STAI, and salivary cortisol on the first and third day after delivery between the four groups (Table 2). However, the score of the PSS on the third day was significantly higher than that on first day in every group (all *p*-value < 0.001) (Table 2). The power of PSS on Day 3 calculated with an effect size 0.258 was 0.74. Moreover, the increasing trend of the PSS in the no-rooming-in group was significantly greater than that in the no-visit group (*p* = 0.02), and the PSS of the rooming-in group was significantly greater than that of the one-visit group (*p* = 0.001) (Fig. 2)

**Table 1:** Demographic data among no-rooming-in, rooming-in, no-visit, one-visit groups

*n*	No-rooming-in 16	Rooming-in 58	No-visit 28	One-visit 47	*P*
Age	33.25 ± 4.48	32.93 ± 4.23	32.46 ± 7.15	33.77 ± 4.56	0.719
Primipara, *n* (%)	6/16 (37.5)	31/58 (53.4)	18/28 (64.3)	30/47 (63.8)	0.233
Education, *n* (%)					
Bachelor	13/16 (81.2)	47/58 (81.0)	22/28 (78.6)	41/47 (87.2)	0.769
Master or above	3/16 (18.7)	11/58 (19.0)	6/28 (21.4)	6/47 (12.8)	
Occupation, *n* (%)					
No	7/16 (43.8)	11/58 (19.0)	4/28 (14.3)	8/47 (17.0)	0.091
Yes	9/16 (56.2)	47/58 (81.0)	24/28 (85.7)	39/47 (83.0)	
Religion, *n* (%)					
No	6/16 (37.5)	21/58 (36.2)	16/28 (57.1)	21/47 (44.7)	0.304
Yes	10/16 (62.5)	37/58 (63.8)	12/28 (42.9)	26/47 (55.3)	

Continuous variables were analyzed by one-way analysis of variance and presented as mean ± standard deviation. Categorical variables were analyzed by the Chi-square or Fisher's exact test and presented as frequency and percentages.

**Table 2:** Perceived stress scale, state-trait anxiety inventory and salivary cortisol among no-rooming-in, rooming-in, no-visit, one-visit groups

*n*	No-rooming-in		Rooming-in		No-visit		One-visit		P*
	16		58		28		47		
		***P*** (95% CI)		***P*** (95% CI)		***P*** (95% CI)		***P*** (95% CI)	
**PSS**									
Day 1 Day 3	22.38 ± 6.31 40.81 ± 13.41	<0.001 (13.95-22.93)	21.72 ± 7.45 42.40 ± 14.51	<0.001 (18.68-22.67)	20.86 ± 6.25 37.40 ± 18.69	<0.001 (6.46-14.83)	22.83 ± 8.39 37.40 ± 18.69	<0.001 (10.74-18.4)	0.715 0.022
**STAIS**									
Day 1 Day 3	29.88 ± 7.41 32.31 ± 10.92	0.332 (-2.74-7.61)	33.93 ± 9.13 36.00 ± 10.79	0.058 (-0.70-4.21)	33.36 ± 8.72 35.36 ± 11.48	0.261 (-1.58-5.58)	36.00 ± 12.39 36.04 ± 12.38	0.964 (-2.77-2.90)	0.207 0.692
**STAIT**									
Day 1 Day 3	38.12 ± 11.53 38.25 ± 12.92	0.951 (-4.12-4.37)	37.26 ±8.66 38.47 ± 9.74	0.083 (-0.16-2.58)	38.86 ± 8.48 36.89 ± 10.16	0.158 (-4.74-0.81)	40.15 ±10.70 39.26 ± 11.35	0.260 (-2.47-0.68)	0.497 0.835
**Salivary cortisol**									
Day 1 Day 3	0.43 ± 0.23 0.50 ± 0.24	0.345 (-0.76-0.20)	0.46 ± 0.26 0.40 ± 0.22	0.078 (-0.13-0.01)	0.49 ± 0.32 0.40 ± 0.16	0.147 (-0.22-0.03)	0.45 ± 0.27 0.47 ± 0.34	0.642 (-0.08-0.12)	0.874 0.322

*P* was calculated by paired t-test between Day 1 and Day 3. P* was calculated by one-way analysis of variance among 4 groups. All data were presented as mean ± standard deviation. *CI* Confidence interval, *PSS* Perceived Stress Scale, *STAIS* State-Trait Anxiety Inventory State, *STAIT* State-Trait Anxiety Inventory Trait

## Discussion

This study is the first to analyze the postpartum stress of Taiwanese mothers who were restricted from visiting their hospitalized children due to the prevention programs of the COVID-19 pandemic. The results showed that mothers’ stress was higher on the third postpartum day than on the first day, regardless of their children’s hospitalization status. Interestingly, the stress levels were still high for mothers whose newborns were healthy and could room-in with their newborns.

As we know, the source of postnatal stress is closely associated with the condition of neonates. According to the Hung postpartum pressure scale, the illness of the neonate is among the top 10 postpartum stressors in Taiwan for primiparous women [24]. Mothers having a newborn admitted to the NICU were a risk factor for maternal postpartum depression and anxiety [10, 25]. Maternal-infant attachment was negatively associated with anxiety and stress symptoms for NICU mothers [9]. Maternal-infant bonding was reported significantly correlated with postpartum depressive symptoms and maternal stress in the early postpartum period, 1-3 days post-delivery [26]. The longer the separation between mother and baby, the greater the mother’s anxiety, which indicates that visiting requirements are essential for reducing mothers’ anxiety associated with hospitalized neonates.

A previous study mentioned that a high prevalence of postnatal psychological distress was associated with the prenatal experience and other individual factors and the pandemic hospital restrictions [27]. Another research also revealed that delivering women during the COVID-19 pandemic had higher postpartum stress than before the COVID-19 pandemic [28]. It should be emphasized that during the COVID-19 pandemic, postpartum mothers experienced a greater degree of psychological stress compared to previously reported stress levels without the extra concern of COVID-19 precautions. In this study, the frequency of visiting was controlled to once a day, and visiting was even prohibited when the pandemic was spreading rapidly. Although most mothers expressed that they fully understood the importance of preventing the spread of COVID-19, they still experienced increasing stress. The stress could be from the policy of visiting restriction or from the COVD-19 disease itself, which was difficult to clearly distinguish. Nevertheless, the COVID-19 pandemic in Taiwan was not severe during most of the study period, which had 400-500 cumulative confirmed cases of COVID-19. People from every walk of life stick to their posts to lift the threat of the pandemic. Life in Taiwan has not much changed, and cities were not locked down. Thus, it is reasonable to estimate that most of the stress was from visiting regulations. According to the increasing trend of PSS in the no-rooming-in mothers (well babies) was greater than that of the no-visit mothers (ill babies) and the increasing trend of the rooming-in mothers (well babies) was also greater than that of the once-a-day visit group (ill babies), it was clear that the stress level from visiting regulations of COVID-19 was significantly greater than that of the stressor of neonatal illness. A study from Australia investigated the childbearing mothers during the COVID pandemic and showed that they felt alone, resulting from physical distancing restrictions, isolation, and needed for society’s support [29]. Besides, women’s partners or support people miss the delivery course and were negatively impacted by restrictions on maternity wards [30]. The rooming-in mothers had the greatest increasing trend that may be caused by a lack of support for baby care.

In the present study, the cortisol values in saliva were not statistically significant regarding the increasing trend of PSS scores, which may have resulted from prenatal stress. This study was initiated at the beginning of COVID-19 in Taiwan when most residents were worried that COVID-19 could cause a high mortality rate, similar to SARS in 2003 [31]. If the cortisol level had increased before delivery, it would negatively affect the hypothalamus-pituitary-adrenal axis activity, which inhibits cortisol secretion after delivery [32]. Additionally, the elevated level of prolactin after delivery would reduce the response of the hypothalamus-pituitary-adrenal axis to stress, and cortisol secretion will also be diminished [33]. Thus, the use of postpartum salivary cortisol to predict postpartum stress may be underestimated. The stress assessment performed before delivery or after the COVID-19 pandemic may be needed to evaluate the changing trend of the stress levels. Furthermore, cortisol level in different tissue samples will react to different types of stress. Blood, saliva, and urine are traditionally used for assessing acute stress (less than 24 hours), and hair and nail can provide chronic stress information (weeks to months) [34]. Further investigation of hair cortisol level may reflect the chronic stress of COVID-19.

## Limitations

The strength of this study was the comparison of mothers’ stress levels between different neonatal states during the pandemic, and the response rate of the questionnaires was 100% because we used web forms to check the answers remotely. This study has several limitations, including the small sample size in the no-rooming-in group. The policy of stopping rooming-in was conducted for only one month in May 2020. During that period, the number of mothers admitted to our hospital for delivery declined because of hospital regulations. Besides, the economic conditions or family members' support didn’t analyze in this study, which could influence the stress of postpartum mothers. In addition, to follow hospital regulations, mothers needed to wear masks when they faced hospital staff, so saliva was hard to collect. Future multiple-centers studies are needed to clarify the long-term impact of COVID-19 on the psychological distress of mothers and their children by using other samples. Additionally, more comprehensive evaluations of the stress-associated symptoms may improve the effect of stress interventions.

## Conclusions

The stress experienced by women who have just given birth appears to be greater in response to the COVID-19 pandemic than that arising from neonatal health. The pandemic has lasted for more than two years, and the end is not in sight. Most specialists focused on preventing the spread of the virus and strictly enforcing the pandemic prevention policy must also help balance the population’s psychological stress. More support is needed during the COVID-19 pandemic to prevent progressive postpartum stress in women after birth.

## Data Availability

The datasets generated and/or analyzed during the current study are not publicly available due to the restriction from academic institutions but are available from the corresponding author on reasonable request.

## Abbreviations

COVID-19: Coronavirus Disease of 2019
HPA: Hypothalamic-pituitary-adrenal
PCR: Polymerase chain reaction
NICU: Neonatal intensive care unit
PSS: Perceived Stress Scale
STAI: State-Trait Anxiety Inventory
